# Case of Fracture of the Skull

**Published:** 1839-04-06

**Authors:** William Zollickoffer

**Affiliations:** Middleburg, (Md.,)


*Case of Fracture of the Skull.* By William Zollickoffer, M. D.

Two years this spring, a messenger came after me in great haste to visit the son of Mr. D. J. Poole. On my arrival at his dwelling, I found Dr. Eichelberger, who had been waiting for me for three hours. The child had been vomiting from the time the injury took place until my arrival. He complained of an insufferable nausea, and pain in the head. On examination, we found a large tumour over the back part of the right parietal bone,—and, on pressure, it was readily ascertained that the part of the skull immediately under the tumour was fractured, and very considerably depressed. In consultation it was concluded, that an imperious duty rendered it obligatory upon us to operate without delay, in order to elevate the depressed bone, or remove it if altogether detached. This task, at the solicitation of Dr. Eichelberger, I performed. On opening the tumour, there was found a large quantity of coagulated blood, that could not escape, in consequence of the skin not having been wounded sufficiently to admit of its passage; after removing it, the superior and posterior portion of the right parietal bone, about the circumference of a dollar, was found to be fractured into four distinct pieces, and driven in upon the substance of the brain at least an inch. Three of these pieces I succeeded in elevating, their superior surfaces being only partially broken, while their inferior superficies were merely bent. The fourth portion I removed, as it was perfectly detached. The *dura* and *pia mater* had sustained considerable lesion in its structural organization, through which a small portion of cerebral matter had made its way. *Immediately* upon elevating and removing the fractured portions, the little sufferer exclaimed—"*Now, my stomach feels quite good, and my head too.*" The skin was now brought into near proximity with the surface of the skull, and secured by a few stitches and adhesive straps, in consociation with a light compress. The subsequent course of therapeutic treatment was strictly antiphlogistic, and modified occasionally to suit the circumstances and exigencies of the case, in order to prevent inflammation of the brain. In three months the wound healed, without the slightest evidence of symptoms of pyrexia. The child, during the compressed condition of the brain, exercised his intellectual faculties as usual. Would not an opposite state of things have been developed, had an injury to the same extent been inflicted on the anterior portion of the head? Attention to the effects resulting from injuries of particular parts of the skull, might have the direct tendency to confirm the views of phrenologists, in the opinion of a plurality of organs, and their connexion with the happy display of the moral and intellectual faculties. The child received the fracture from falling, while in the act of climbing in the barn, with his head upon a large stone. This was his report of the manner in which the accident took place.

*Middleburg*, (*Md.*,) April 1st, 1839.

